# Deep Learning-Based Recognition of Different Thyroid Cancer Categories Using Whole Frozen-Slide Images

**DOI:** 10.3389/fbioe.2022.857377

**Published:** 2022-07-06

**Authors:** Xinyi Zhu, Cancan Chen, Qiang Guo, Jianhui Ma, Fenglong Sun, Haizhen Lu

**Affiliations:** ^1^ Department of Pathology, National Cancer Center/National Clinical Research Center for Cancer / Cancer Hospital, Chinese Academy of Medical Sciences and Peking Union Medical College, Beijing, China; ^2^ Digital Health China Technologies Corporation Limited, Beijing, China; ^3^ Department of Big Data, National Cancer Center/National Clinical Research Center for Cancer / Cancer Institute and Hospital, Chinese Academy of Medical Sciences and Peking Union Medical College, Beijing, China; ^4^ Department of Urology, National Cancer Center/National Clinical Research Center for Cancer / Cancer Institute and Hospital, Chinese Academy of Medical Sciences and Peking Union Medical College, Beijing, China

**Keywords:** thyroid cancer, pathology, WSI, rare category, deep learning model

## Abstract

**Introduction:** The pathological rare category of thyroid is a type of lesion with a low incidence rate and is easily misdiagnosed in clinical practice, which directly affects a patient’s treatment decision. However, it has not been adequately investigated to recognize the rare, benign, and malignant categories of thyroid using the deep learning method and recommend the rare to pathologists.

**Methods:** We present an empirical decision tree based on the binary classification results of the patch-based UNet model to predict rare categories and recommend annotated lesion areas to be rereviewed by pathologists.

**Results:** Applying this framework to 1,374 whole-slide images (WSIs) of frozen sections from thyroid lesions, we obtained an area under a curve of 0.946 and 0.986 for the test datasets with and without WSIs, respectively, of rare types. However, the recognition error rate for the rare categories was significantly higher than that for the benign and malignant categories (*p* < 0.00001). For rare WSIs, the addition of the empirical decision tree obtained a recall rate and precision of 0.882 and 0.498, respectively; the rare types (only 33.4% of all WSIs) were further recommended to be rereviewed by pathologists. Additionally, we demonstrated that the performance of our framework was comparable to that of pathologists in clinical practice for the predicted benign and malignant sections.

**Conclusion:** Our study provides a baseline for the recommendation of the uncertain predicted rare category to pathologists, offering potential feasibility for the improvement of pathologists’ work efficiency.

## Introduction

Thyroid cancer is one of the most common cancers worldwide, which ranks seventh among females in the United States (Siegel et al., 2021) and fifth in China (Zhang et al., 2021). The 2021 cancer statistics report released by the American Cancer Society (ACS) shows that the incidence of thyroid cancer is about 14.1/100,000 people, accounting for 93.8% of all endocrine system malignancies (Siegel et al., 2021). In 2022, according to the latest statistics on the national tumor situation in 2016, the National Cancer Center concluded that the incidence of thyroid cancer in China was 202,600, and the incidence rate was 14.65/100,000 people (Zheng et al., 2022). Its occurrence has been increasing in recent years (Seib and Sosa, 2019; Zheng et al., 2022). Several guidelines or consensus have been established for treating thyroid cancer (Haugen et al., 2016; Filetti et al., 2019; Tuttle et al., 2019; Ozgur et al., 2021), which depends on the accurate pathological diagnosis of the disease. For example, the intraoperative frozen section (FS) diagnosis is crucial in determining the surgical strategy for thyroid cancer treatment. The 2015 American Thyroid Association management guideline recommendation also affirmed the significance of intraoperative FSs in diagnosing classical papillary thyroid cancer (PTC) (Haugen et al., 2016). However, rare tumor types and unevenly processed specimens that can produce artifices can present a challenge to the onsite pathologists, and they may have to defer the intraoperative diagnosis to the paraffin section. The most common type of thyroid is PTC (∼83.6–98.2%), which is easy to diagnose in most cases. The rare subtypes are difficult to diagnose using hematoxylin and eosin staining, and they include the following: follicular thyroid carcinoma (∼0.9–10.8%), medullary thyroid cancer (approximately 0.6–2.2%), and undifferentiated carcinoma (approximately 0.1%) (Lim et al., 2017; Zhao et al., 2019; He and Wei, 2021). Similar phenomena exist in lung cancer (de Sousa and Carvalho, 2018; Shirsat et al., 2021) and breast cancer (Jenkins et al., 2021), among others. The pathological image data from the rare categories are difficult to obtain because of their low incidence rate, which results in low diagnostic consistency among pathologists and improperly building a convolutional neural network (CNN) model directly. Thus, recognizing the rare (or intermediate) categories (Adamson and Welch, 2019) is a special and inevitable question in the field of computational pathology domain.

Based on the wide application of deep learning in industries, such as convolutional neural networks (CNNs) and recurrent neural networks (RNNs), several excellent studies have been conducted to develop computer-aided diagnostic systems for histopathology (Zhang et al., 2019; Skrede et al., 2020). The developed digital pathology technology provides a basis for using deep learning algorithms in histopathological diagnoses. The Cancer Metastases in Lymph Nodes Challenge 2016 (CAMELYON16) (https://camelyon16.grand-challenge.org) and the public whole-slide image (WSI) dataset from The Cancer Genome Atlas (TCGA) (https://www.cancer.gov/about-nci/organization/ccg/research/structural-genomics/tcga) significantly promote the implementation of patch-based CNNs for WSIs in cancer histopathology; this is to verify the feasibility of CNN methods for the diagnosis of lymph nodes’ metastases in breast cancer. The area under the curve (AUC) of the InceptionV3 (Szegedy et al., 2016) model using the CAMELYON16 dataset is 98.6%, and the free-response receiver operating characteristic (FROC) curve is 87.3% (Szegedy et al., 2016; Liu et al., 2017). The Resnet model and a conditional random field (Li and Ping, 2018) were used to exploit the context information of patch images in WSIs, and the corresponding FROC curve using CAMELYON16 is 79.34%. Moreover, CNNs have been trained and evaluated in other cancer categories. For example, the InceptionV3 model was validated using TCGA non-small cell lung cancer histopathology images (Coudray et al., 2018), and the study reported that the performance of the developed framework did not show a statistically significant difference compared with three pathologists (two and one thoracic and anatomic pathologist, respectively). Additionally, to address the interpretability of the deep learning model for cancer diagnosis, a novel pathology WSI diagnostic method was developed in urothelial carcinoma of bladder cancer and compared with 17 pathologists to verify the diagnostic accuracy of the framework (Zhang et al., 2019). Furthermore, several general classifications and segment models, such as MobileNet (Howard et al., 2017) and UNet (Ronneberger et al., 2015), were developed for prostate cancer, basal cell carcinoma, and colorectal cancer (Campanella et al., 2019; Skrede et al., 2020). These studies jointly demonstrate the significant potential of CNNs in computer-aided diagnostic systems for histopathology.

Most of the previous results from deep learning-based studies focused on WSIs from common benign and malignant subtypes without considering the rare ones. The model based on data from only the common subtypes cannot function efficiently to predict the rare ones. In clinical settings, the diagnostic results of pathological images are used to guide the selection of operation, which requires high accuracy for both the common and the rare subtypes. However, we do not know if the next section to be evaluated is from a common or rare subtype. Thus, the model prediction results are impractical. In this study, we collected 1,374 thyroid FSs at the National Cancer Center/Cancer Institute and Hospital, and the Chinese Academy of Medical Sciences (NCC/CICAMS) from September 2018 to December 2020. We developed a novel framework to effectively automate whole-slide diagnosis and classification into three categories based on the dataset: common benign, common malignant, and rare categories. Our AUC for binary classification using the patch-UNet model was approximately 0.986 for WSIs obtained from common benign and malignant tumors, whereas the AUC was only 0.946 when the rare category was included. The use of an empirical decision tree and the patch-UNet model obtained a 0.882 recall rate (127/144 WSIs) for the rare types and resulted in 33.4% of WSIs (255 WSIs) from the entire test dataset being rereviewed by pathologists.

## Materials and Methods

### Dataset

The NCC Ethics Committee/Institution Review Board (2021031709490902) approved our research. In this study, patient consent was not required as participants were not at risk. We collected 1,374 thyroid FSs in the Pathology Department of NCC/CICAMS from September 2018 to December 2020, which includes the following: 536 PTC, 72 thyroid adenomatous lesions (TAL), 45 thyroid fibrous calcified nodules (TFCN), 691 nodular goiters (NG), 5 other thyroid carcinomas (Other TC: which includes medullary thyroid carcinoma, undifferentiated carcinoma, and poorly differentiated carcinoma), and 25 other benign thyroid lesions (Other BTL: which includes thyroiditis and granulomatous thyroiditis) (Table 1). To generate the WSIs, all FSs were scanned using the Aperio AT2 Digital Whole Slide Scanning System (Leica Biosystems, Germany). Notably, we have excluded images containing artifacts of tissue processing, including air bubbles, folding, handwriting, crushing, poor staining, and blurring when scanning the sections, which seriously affected the recognition.

**TABLE 1:** Dataset information.

Ground truth	Common/rare	Subtype	WSI count	Percentage (%)
Malignant	Common	PTC	536	39.01
Malignant	Rare	Other TC	5	0.36
Intermediate	Rare	TAL	72	5.24
Intermediate	Rare	TFCN	45	3.28
Benign	Rare	Other BTL	25	1.82
Benign	Common	NG	691	50.29
Total	—	—	1374	100

### Reference Standard

In the clinical practice, each FS was reviewed by two additional pathologists, and the diagnostic report was given and deposited in the pathology reports system. Generally, the so-called intermediate categories are indeed prone to misdiagnosis, but it does not mean that their biological behavior is unclear. We collected and reevaluated all reports and then grouped them as malignant, benign, and intermediate. The malignant group included PTC and Other TC, benign included NG and Other BTL, and intermediate included TAL and TFCN. The lesion areas of all PTC, other TC, TAL, and TFCN were annotated on digital slides manually by four pathologists with over two years of work experience who trained before the annotation using Bejnordi et al.‘s method (Ehteshami Bejnordi et al., 2017) and ASAP software (V1.9.0, https://github.com/computationalpathologygroup/ASAP/releases). Each annotated WSI was reviewed in detail by a pathology expert with over 20 years of work experience.

### Dataset Splitting

Our dataset was randomly split at the WSI level into 496 slides for training (36%), 114 slides for validation (8%), and case series (Test 1 total (45%), 617; Test 2 total (56%), 764) for testing (Table 2). The training and validation sets contained the PTC and NG WSIs. However, in the test group, the Test 1 dataset contained common benign and malignant pathological subtypes (PTC and NG) commonly used in research. To evaluate the performance of the commonly used patch-UNet model in clinical practice, we designed the Test 2 dataset containing all subtypes (PTC and NG) in Test 1 and the rare category. So the Test 2 dataset contained not only PTC and NG types included in Test 1 but also two intermediate cases that are also known as rare types (TAL and TFCN); it also contained two other rare types (Other TC and BTL). Thus, the composition of Test 2 simulated a real-world dataset in clinical practice.

**TABLE 2:** WSI counts for training validation and test datasets.

Ground truth	Subtype	Training	Validation	Test1^a^	Test2^a^
Malignant	PTC	200	53	283	283
Malignant	Other TC	0	0	0	5
Intermediate	TAL	0	0	0	72
Intermediate	TFCN	0	0	0	45
Benign	Other BTL	0	0	0	25
Benign	NG	296	61	334	334
Total	—	496	114	617	764

a Test 1 simulates the dataset for research. Test 2 simulates the real-world dataset in clinical practice.

### Patch Sampling

**FIGURE 1:**
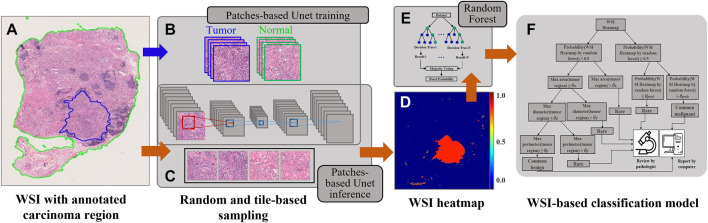
An overview of the proposed WSI diagnostic framework presented in this study. **(A)** The WSI slide with the region of interest (green line) and carcinoma region (blue line). **(B)** The process of patch-based UNet model training. **(C)** The process of patch-based UNet inference. **(D)** The WSI heatmap. **(E)** A random forest was selected for the WSI-based classification task. **(F)** The proposed triple classification model.

Our framework was based on WSIs divided into patches because of their large size (over 10 gigapixels at 40 × equivalent magnification). First, we used Otsu’s method (Gharib et al., 2010) to remove empty regions in WSIs, which significantly lowered the computational cost. To avoid biases toward training slides, WSIs were randomly sampled using a 256 × 256 resolution (at 5 × equivalent magnification) and patches were extracted from every WSI with an equal number (Figure 1B). We performed a set of ratios (1:1, 1:2, 1:3, and 1:4) for sampling malignant and benign patches on our dataset, and the maximum accuracy on the Test 1 dataset was used to decide the best condition in each experiment. The ratio accuracy 1:1, 1:2, 1:3, and 1:4 was 0.9449, 0.9627, 0.9643, and 0.9559, respectively. The 1:3 ratio achieved the best results over the other tested ratios. To reduce the false positives, the ratio of malignant and benign patches adopted was 1:3 (Liu et al., 2019).

### Patch-UNet Model Training and Inference

The training set of the UNet model was constructed using the aforementioned malignant and benign patches, and similarly, we obtained a validation set. For training, we used softmax cross-entropy as the loss function, exponential decay mechanism for learning rate management, batch size of 32, and an initial learning rate of 0.01 based on empirical values to reduce invalid trials and computational consumption. The UNet model was trained without a pretrained model until convergence occurred in both the training and test datasets.

During UNet inference, all WSIs were partitioned into 512 × 512 images in equally spaced intervals using a stride of 256 × 256 to generate WSI heatmaps, which is known as the tile-based sampling (Figure 1C). Thus, the core size 256 × 256 of 512 × 512 images was used to compose the heatmap, and other marginal edges were abandoned to remove the edge noise of 512 × 512 images. Furthermore, as a fully convolutional network, UNet allows an equal ratio of scaled sizes between training and inference.

### Postprocessing of the Heatmap for Binary and Triple Classification

We applied a random forest as the two slide-level classification models because the mean accuracy of different pathological subtypes by using a random forest (0.7712) was higher than that by SVM (0.4751). Furthermore, we extracted four types of features from heatmaps (Wang et al., 2016; Wen et al., 2017) (Figure 1E), such as geometric features (area, perimeter, eccentricity, extent, and solidity), texture features (gradient and intensity), marginal features (canny nonzero and mean), and others (such as connected region and pixel count). The statistics consist of the maximum, minimum, mean, standard deviation, variance, skewness, kurtosis, entropy, energy, contrast, dissimilarity, homogeneity, and correlation. Based on these features, we trained the random forest model for WSI binary classification. All features are listed in Supplementary Table S1, and the corresponding feature is presented in Supplementary Figure S1 (Supplementary Material). AUC was calculated and used to evaluate the model’s performance. PTC, TAL, TFCN, and other TC WSIs were considered positive, whereas other BTL and NG slides were negative.

The important features selection during RF model training was as follows. First, we trained the random forest with all the features (Supplementary Table S1) and obtained the top 30 most important features. Then, these features were used to train another RF model to provide the binary-classification result for each WSI. The top 30 most important features could be summarized into four categories: lesion count, lesion area related, diameter related, and perimeter (shape) related (Supplementary Figure S1 and Supplementary Table S2). These features can also provide crucial evidence for pathologists’ decision-making in clinical practice. When multiple scattered lesions were observed, it revealed that malignant lesions had occurred. However, when the lesions are diffusely distributed, the possibility of aggressively malignant tumors or thyroiditis should be considered. Furthermore, when the lesion area or diameter is larger, the tumor cell components are more complex, and the probability of malignant components would increase. Moreover, malignant lesions show obvious irregular edges, resulting in a relatively larger lesion perimeter. However, benign thyroid tumors, such as TAL, TFCN, and NG, have relatively regular and round edges without invasive growth characteristics, resulting in relatively smaller lesion perimeter (Chmielik et al., 2018).

To build the triple classification model, we assumed the features will be easier to qualify, explain, and visualize in clinical applications for future computer-aided diagnosed product development. Thus, the probability of the binary random forest classifier, the carcinoma diameter, perimeter, and area were used to design the triple classification model. We designed the postprocessing steps as follows using the Test 2 dataset. The probability of the heatmap computed by random forest was the first parameter. The next includes the max area, diameter, and perimeter of tumor regions on WSI heatmaps. All WSIs were sequentially split into two sets using the aforementioned features. The corresponding division values for probability, area, diameter, and perimeter were 0.5, 300 mm^2^, 7 mm, and 35 mm, respectively (Figure 4 and Figure 1F). To evaluate the triple classification result, we divided the Test 2 dataset into three categories: common benign, common malignant, and rare. The common benign and malignant groups represent the benign (NG) and malignant (PTC) types, respectively, which are correctly classified using patch-UNet. The rare category represents the misclassified PTC, other TC, hard determined NG and other BTL, and intermediate WSIs (Table 3). For the triple classification results, WSIs predicted as common benign and malignant could be diagnosed using our model, but the others must be rereviewed by pathologists. We first divided the Test 2 dataset into training and test sets (Supplementary Table S3), and we trained a triple-classification model using a random forest. However, we observed that the recall rate for the rare category is only 0.743, which is lower than the performance of our empirical tree (0.882).

**TABLE 3:** The test dataset in clinical practice was relabeled based on our binary classification results and the ground truth for evaluating the triple classification model performance.

Subtype	The common benign^a^	The common malignant^a^	The rare^a^	Total
PTC	0	270	13	283
Other TC	0	5	0	5
TAL	0	0	72	72
TFCN	0	0	45	45
Other BTL	20	0	5	25
NG	325	0	9	334
Total	345	275	144	764

a The common benign and malignant represented the types correctly classified by patch-UNet, for the benign (NG) and malignant (PTC) types, respectively. The rare represented the misclassified PTC, other TC, hard determined NG, other BTL, and the intermediate WSIs.

## Results

### Binary Classification

**FIGURE 2:**
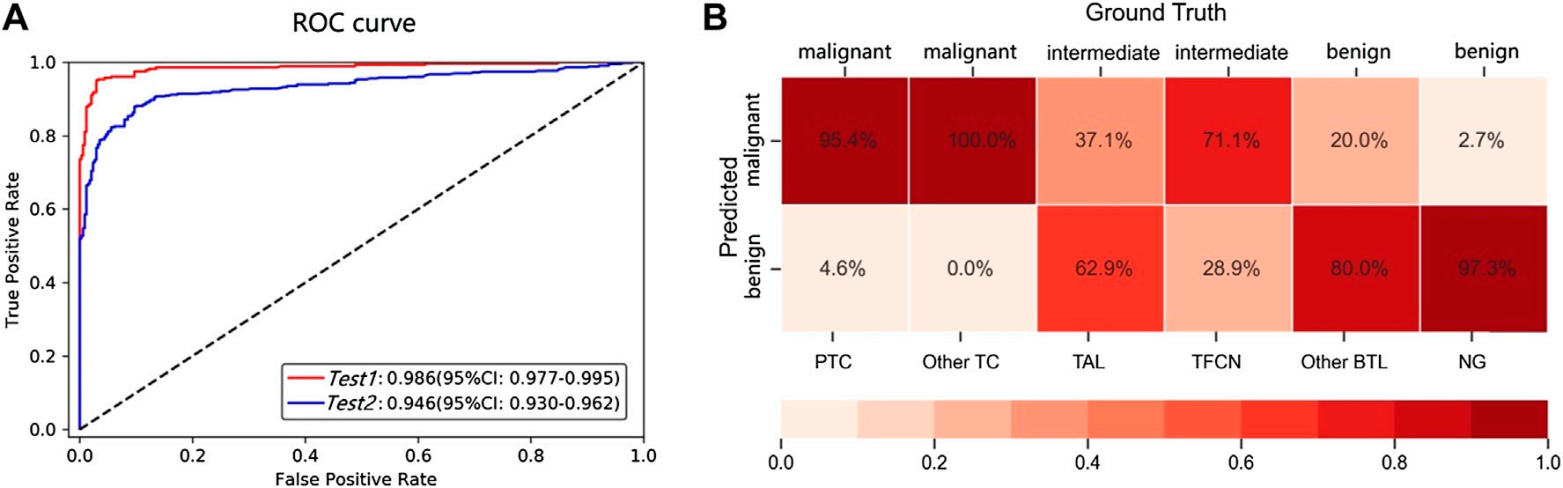
**(A)** AUC for the different test datasets. The Test 1 dataset contained common benign and malignant pathological subtypes (PTC and NG). Test 2 contained not only the common PTC and NG types included in Test 1 but also two intermediate types (TAL and TFCN) and two other rare types (other TC and BTL). **(B)** The confusion matrix for the benign, malignant, and intermediate subtypes.

We conducted experiments to evaluate whether the performance of the patch-UNet model was sufficient for clinical practice (Figure 1). To simulate the real clinical setting in these experiments, we designed three test groups of different sizes (Test 1 total, 617; Test 2 total, 764) using common training and validation sets (Materials and Methods, Table 2). Our AUC for binary classification was approximately 0.986 for Test 1, which corresponds to the model performance in the research theory. However, when the same model was applied to the Test 2 dataset containing the rare category, the AUC was 0.946, indicating a decrease of 0.04 compared with its performance in the Test 1 dataset (Figure 2A). Additionally, we also obviously drop for specificity and positive predicted value (PPV) (Supplementary Table S5). These results showed that the current patch-UNet model trained with FSs from the easily collected pathologic types of thyroid cancer could not maintain its performance in clinical practice.

### Misclassification

**FIGURE 3:**
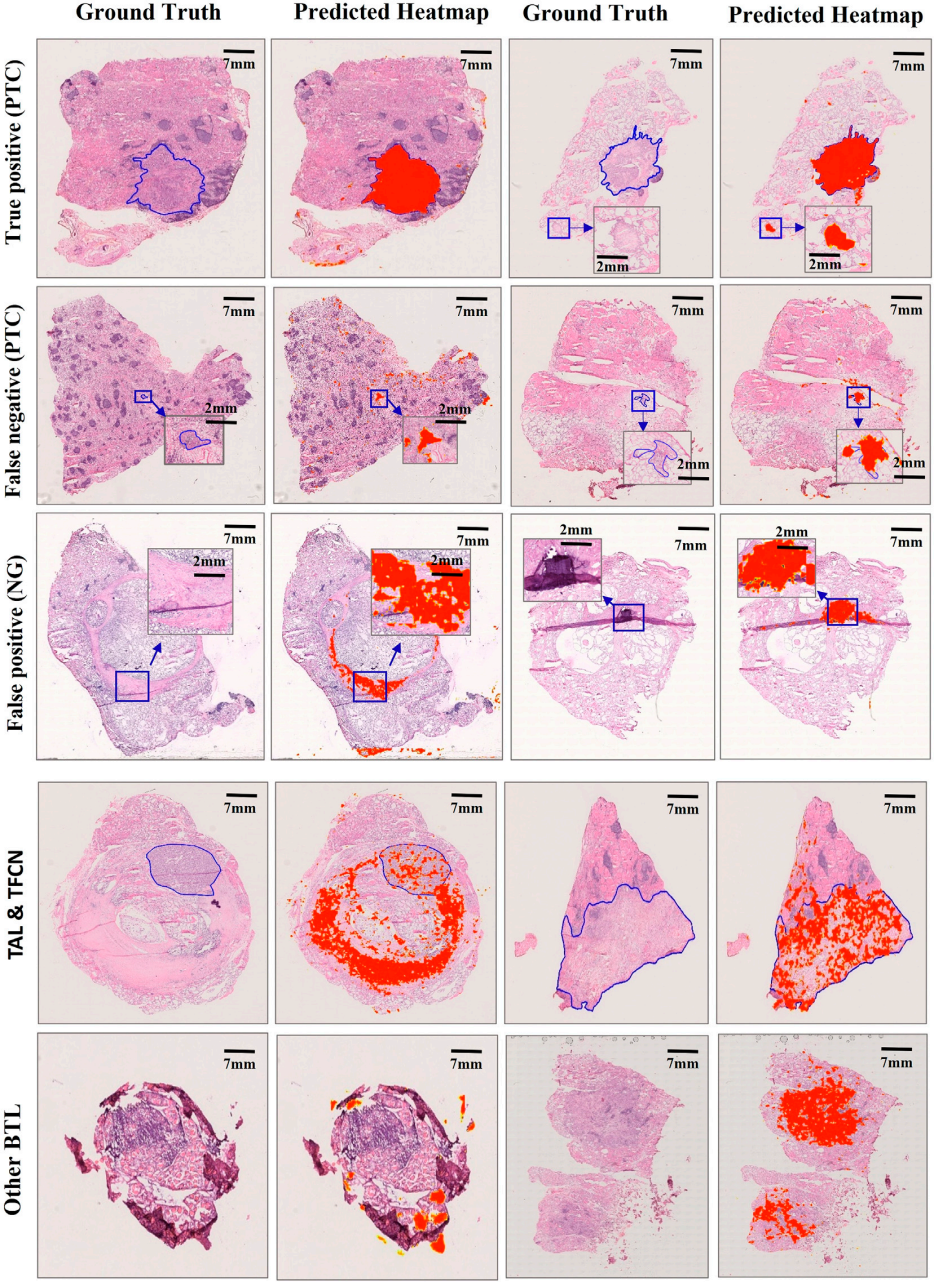
Misclassification examples of selected slides in the Test 1 dataset. Examples of true positive, false negative, false positive, and other subtype slides are represented. Our model performed efficiently in tumor carcinomas for true positives (PTC), but the false positives (NG) were mainly caused by the fibrotic tissue in both the normal and carcinoma regions. Fibrotic tissue sometimes has a larger area or diameter than certain carcinoma regions for false negatives (PTC). Because fibrotic tissue is quite common in thyroid WSIs, the TAL, TFCN, and other BTL slides outside our training slides showed clear differences in structural features from PTC and NG slides, resulting in a heatmap far from the ground truth.

The following analysis was conducted to determine the reason for the decreased AUC value when the model was used for the Test 2 dataset. We observed that the total number of misdiagnosed WSIs was 84, which includes 14 and 70 false positives and negatives, respectively. However, if the WSIs of intermediate types are removed from the Test 2 dataset, the error predicted number is only 22, which includes 9 and 13 false positives and negatives, respectively (Figure 3). Furthermore, we observed that the error rate for the intermediate types was as high as 48.7% (Figure 2B), which was significantly higher than the benign and malignant categories (*p* < 0.00001). To further understand the reason for this occurrence, an experienced pathologist and an artificial intelligence specialist rereviewed all 84 error-predicted WSIs. The fibrotic tissue in both tumors and noncancerous regions of WSIs resulted in the misclassification of false-positive slides. Furthermore, the cell and structural features of the intermediate category varied from the slides of the training set, thus reducing the accuracy of the model for the intermediate category (Figure 3). However, clinical thyroid slides of different subtypes always have substantial diverse features and distributions. These findings indicate that using the single two-classification patch-UNet method for the clinical application of computer-aided diagnosis for thyroid cancer WSIs was impractical.

### Triple Classification

**FIGURE 4:**
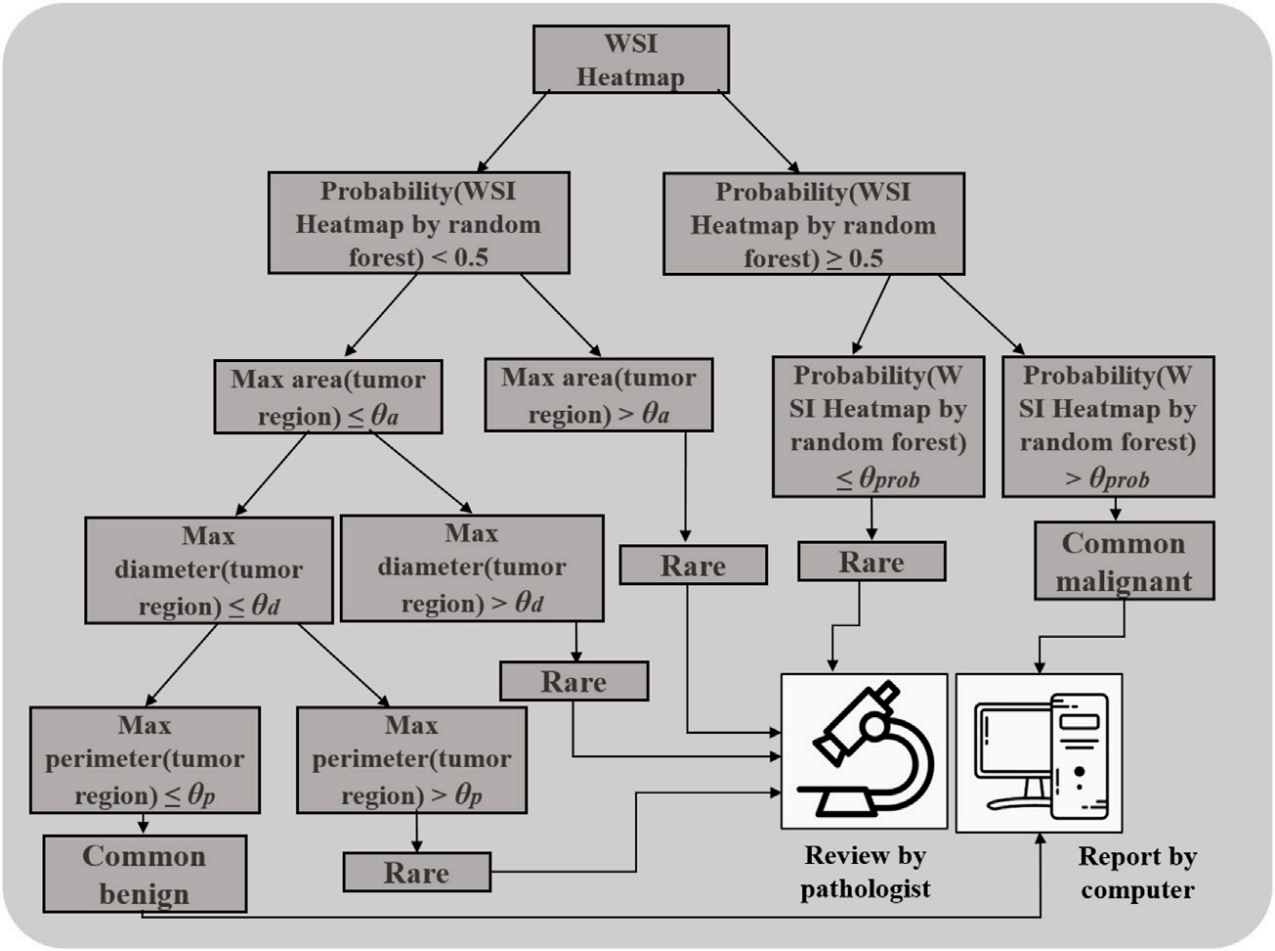
Decision tree for distinguishing the rare from the common benign and malignant categories.

To limit the effect of the decreased AUC in clinical practice, we first relabeled the WSIs from the complete test dataset based on the prediction results of the patch-UNet model and the subtype distributions, which include the rare, common benign, and malignant categories (Materials and Methods, Table 3). To distinguish the rare category, we designed a decision-tree model (Figure 4). For the rare category, we obtained a recall rate and precision of 0.882 and 0.498, respectively, and we recommended that they are rereviewed by pathologists. Notably, all predicted intermediate images accounted for 33.4% of WSIs (255 WSIs) from the Test 2 dataset, whereas the rest (509 WSIs) could be diagnosed directly using our models. To compare the accuracy of our model for 509 WSIs with that of pathologists in clinical practice, we collected the reports from the pathology report system of CICAMS and observed that eight FSs were misdiagnosed. However, there was no significant difference between our model’s performance and that of pathologists in clinical practice using the Fisher and chi-square test (*p* > 0.05, Supplementary Table S4).

## Discussion

The rare types are inevitable, and the special question of pathology image recognition is not a well-investigated research area. The main hypothesis of this research was that we could achieve the required accuracy level for most FSs from thyroid cancer in clinical practice by recognizing the rare types between common benign and malignant cases of WSIs. To test this hypothesis, we first performed a binary classification using patch-UNet and then used an empirical decision tree for triple classification. Furthermore, we demonstrated that our framework performed at the level expected by pathologists in clinical practice for the common benign and malignant sections predicted. In clinical practice, these three types correspond to different clinical treatments. When the model suggests an intermediate category, the pathologist needs to review it. Otherwise, misdiagnosis is likely to occur, which affects treatment decisions. In addition, our framework might theoretically handle 66.6% of the diagnostic workload of thyroid pathologists.

The incidence rate of thyroid cancer pathological subtypes is different (Lim et al., 2017; Zhao et al., 2019; He and Wei, 2021). Thus, the proportion of PTC and NG WSIs in our dataset was approximately 90%, whereas that of the other types (TAL, TFCN, other TC, and Other BTL) was significantly lower at ∼10%, resulting in an insufficient number of WSIs for deep learning model training. Previous studies have obtained encouraging results for prostate cancer (AUC, 0.986), skin cancer basal cell carcinoma (AUC, 0.986) (Campanella et al., 2019), bladder cancer (AUC, 0.95) (Zhang et al., 2019), gastric cancer (sensitivity, approximately 100%; specificity, 80.6%) (Song et al., 2020), lung cancer (AUC >0.974) (Kanavati et al., 2020), and cervical cancer (AUC, 0.978) (Tian et al., 2019). However, most of these models were generated based on the main cancer type. This limits their applications because, in clinical practice, machines do not know whether the next section to be reviewed is from a common or rare subtype. In our study, we not only achieved high classification performance with an AUC of 0.986 but also improved the recognition of the rare category (recall, 0.882; precision, 0.498), and we further recommended these sections be rereviewed by pathologists. Additionally, an improved region growing algorithm has a better segmentation effect (Li et al., 2019), providing insight for tumor-region detection at WSI-level and may further improve the performance of the recognition of the rare category.

Unlike the commonly used image datasets, such as ImageNet, tumor pathological images lack a ground truth for the gray zone (intermediate category) between cancer and non-cancer. Consequently, the recognition of the intermediate category between cancer and non-cancer should be considered seriously before artificial intelligence technology is widely applied (Adamson and Welch, 2019). TAL and TFCN are mostly benign thyroid lesions in our study. However, during the pathological diagnosis process, they do not belong to the common benign thyroid (NG) or malignant nodules (PTC), and they must be occasionally discussed and disputed during the diagnostic process. The former should be differentiated with encapsulated follicular-patterned thyroid tumors newly added by WHO in 2017 (Lloyd et al., 2017), whereas the latter should be differentiated with papillary thyroid carcinoma with abundant calcification. These lesions can be diagnosed only after thorough observation and taking sufficient specimens. Some cases require time to demonstrate their biological behavior (Rosario and Mourão, 2019). Thus, TAL and TFCN were temporarily classified as intermediate lesion groups. In this study, we expanded the intermediate’s definition using WSIs from the benign and malignant categories that were misclassified by our patch-UNet model, which is known as the rare type.

Because there is a small proportion of rare types of thyroid lesions in practice, the rare types collected during the experiment are insufficient for deep learning. Thus, when simulating the diagnosis of thyroid lesions in the real clinical setting, the model is more prone to misclassification of the rare types. We assume that misclassification occurs mainly because there are similarities in pathological features between benign and malignant thyroid lesions to some extent (Dov et al., 2021). For example, at the cellular level, Hashimoto’s thyroiditis may have obvious follicular epithelial hyperplasia (enlarged nuclei, crowded cells, etc.) and mild nuclear atypia (chromatin margins, nuclear membrane irregularities, etc.), which are confused with malignant lesions; cell enlargement can also be seen locally in benign thyroid tissue (Girolami et al., 2020; Böhland et al., 2021). Benign thyroid tissue may also have a background of fibrosis and calcification at the background level, which is common in thyroid cancer. The mode will recall rare types and submit them to the pathologist for review. Furthermore, our method efficiently combines deep learning technology with the clinical requirements for the diagnosis of pathological sections.

This study has some limitations. First, our dataset was collected without considering all the rare types equally and lacked external validation, for example, we only collected 5 WSIs from other TC, while 72 WSIs from TAL (Table 1). However, we merged WSIs from other TC, TAL, TFCN, and other BTL together for algorithm development. Actually, it is very hard to collect enough WSI, especially the rare ones for external validation, so we first demonstrated our framework is technically feasible for the question raised in this study. Second, the study is based on the histopathological review of thyroid WSIs in CICAMS, however, there is no general quality control standard for the production process of pathological sections and WSIs (Webster and Dunstan, 2014; Aeffner et al., 2019), which will have a significant impact on the model generality. In the future, one feasible strategy is that each institution develop its image analysis algorithms and internally validated by before the algorithms can be used for clinical care (Aeffner et al., 2019).

In summary, we described a deep learning method for diagnosing different types of thyroid cancer using WSIs for the first time. Furthermore, we focused on the intermediate category and demonstrated the feasibility of our model from a clinical application perspective. We expect that our experimental design and method may be invaluable for the thyroid cancer diagnosis and significantly improve the application of deep learning methods for other types of cancer. Because of the further maturity of this method, fine-tuning the empirical decision tree in a clinical setting is the priority; this machine-and-manual model may ensure diagnostic accuracy, improve diagnostic efficiency, and relieve psychological pressure on pathology experts. Furthermore, we want to initiate random prospective non-interventional clinical trials using this technology to test its efficiency and further advance the application of artificial intelligence in histopathology.

## Data Availability

The data are not publicly available due to hospital regulations. However, data requests with aims will be needed to assess the reasonability. After approval from the hospital and the corresponding authors, de-identified clinical data will be provided. Requests to access the datasets should be directed to luhz@cicams.ac.cn.

## Abbreviations

FS, frozen section; NG, nodular goiter; Negative, other BTL and NG WSIs were considered negative; other BTL, other benign thyroid lesions (including thyroiditis and granulomatous thyroiditis in our research); PTC, papillary thyroid cancer; positive, PTC, TAL, TFCN, and other TC WSIs were considered positive; TAL, thyroid adenomatous lesion; the rare category, other TC, other BTL, TAL, TFCN, misclassified PTC and hard determined NG; other TC, other thyroid cancers (including medullary thyroid carcinoma, undifferentiated carcinoma and poorly differentiated carcinoma in our research); TFCN, thyroid fibrous calcified nodule; note, the magnification of microscope in the manuscript refers to the objective lens.

## References

[B1] Adamson A. S. Welch H. G. (2019). Machine Learning and the Cancer-Diagnosis Problem - No Gold Standard. N. Engl. J. Med. 381 (24), 2285–2287. 10.1056/NEJMp1907407 31826337

[B2] Aeffner F. Zarella M. D. Buchbinder N. Bui M. M. Goodman M. R. Hartman D. J. (2019). Introduction to Digital Image Analysis in Whole-Slide Imaging: A White Paper from the Digital Pathology Association. J. Pathology Inf. 10, 9. 10.4103/jpi.jpi_82_18 PMC6437786 30984469

[B3] Böhland M. Tharun L. Scherr T. Mikut R. Hagenmeyer V. Thompson L. D. R. (2021). Machine Learning Methods for Automated Classification of Tumors with Papillary Thyroid Carcinoma-Like Nuclei: A Quantitative Analysis. PLoS One 16 (9), e0257635. 10.1371/journal.pone.0257635 34550999 PMC8457451

[B4] Campanella G. Hanna M. G. Geneslaw L. Miraflor A. Werneck Krauss Silva V. Busam K. J. (2019). Clinical-Grade Computational Pathology Using Weakly Supervised Deep Learning on Whole Slide Images. Nat. Med. 25 (8), 1301–1309. 10.1038/s41591-019-0508-1 31308507 PMC7418463

[B5] Chmielik E. Rusinek D. Oczko-Wojciechowska M. Jarzab M. Krajewska J. Czarniecka A. (2018). Heterogeneity of Thyroid Cancer. Pathobiology 85 (1-2), 117–129. 10.1159/000486422 29408820

[B6] Coudray N. Ocampo P. S. Sakellaropoulos T. Narula N. Snuderl M. Fenyö D. (2018). Classification and Mutation Prediction from Non-Small Cell Lung Cancer Histopathology Images Using Deep Learning. Nat. Med. 24 (10), 1559–1567. 10.1038/s41591-018-0177-5 30224757 PMC9847512

[B7] de Sousa V. M. L. Carvalho L. (2018). Heterogeneity in Lung Cancer. Pathobiology 85 (1-2), 96–107. 10.1159/000487440 29635240

[B8] Dov D. Kovalsky S. Z. Assaad S. Cohen J. Range D. E. Pendse A. A. (2021). Weakly Supervised Instance Learning for Thyroid Malignancy Prediction from Whole Slide Cytopathology Images. Med. Image Anal. 67, 101814. 10.1016/j.media.2020.101814 33049578 PMC7726041

[B9] Ehteshami Bejnordi B. Veta M. Johannes van Diest P. van Ginneken B. Karssemeijer N. Litjens G. (2017). Diagnostic Assessment of Deep Learning Algorithms for Detection of Lymph Node Metastases in Women with Breast Cancer. JAMA 318 (22), 2199–2210. 10.1001/jama.2017.14585 29234806 PMC5820737

[B10] Filetti S. Durante C. Hartl D. Leboulleux S. Locati L. D. Newbold K. (2019). Thyroid Cancer: ESMO Clinical Practice Guidelines for Diagnosis, Treatment and Follow-Up. Ann. Oncol. 30 (12), 1856–1883. 10.1093/annonc/mdz400 31549998

[B11] Gharib H. Papini E. Paschke R. Duick D. S. Valcavi R. Hegedüs L. (2010). American Association of Clinical Endocrinologists, Associazione Medici Endocrinologi, and European Thyroid Association Medical Guidelines for Clinical Practice for the Diagnosis and Management of Thyroid Nodules: Executive Summary of Recommendations. J. Endocrinol. Invest. 33 (5 Suppl. l), 287–291. 10.1007/bf03346587 20479572

[B12] Girolami I. Marletta S. Pantanowitz L. Torresani E. Ghimenton C. Barbareschi M. (2020). Impact of Image Analysis and Artificial Intelligence in Thyroid Pathology, with Particular Reference to Cytological Aspects. Cytopathology 31 (5), 432–444. 10.1111/cyt.12828 32248583

[B13] Haugen B. R. Alexander E. K. Bible K. C. Doherty G. M. Mandel S. J. Nikiforov Y. E. (2016). 2015 American Thyroid Association Management Guidelines for Adult Patients with Thyroid Nodules and Differentiated Thyroid Cancer: The American Thyroid Association Guidelines Task Force on Thyroid Nodules and Differentiated Thyroid Cancer. Thyroid 26 (1), 1–133. 10.1089/thy.2015.0020 26462967 PMC4739132

[B14] He J. Wei W. (2021). 2019 China Cancer Registry Annual Report. Beijing: People's Medical Publishing House.

[B15] Howard A. G. Zhu M. Chen B. Dmitry K. Wang W. Tobias W. (2017). MobileNets: Efficient Convolutional Neural Networks for Mobile Vision Applications. ArXiv e-prints [Preprint]. Available at: https://arxiv.org/abs/1704.04861 (Accessed April 17, 2017).

[B16] Jenkins S. Kachur M. E. Rechache K. Wells J. M. Lipkowitz S. (2021). Rare Breast Cancer Subtypes. Curr. Oncol. Rep. 23 (5), 54. 10.1007/s11912-021-01048-4 33755810 PMC8204849

[B17] Kanavati F. Toyokawa G. Momosaki S. Rambeau M. Kozuma Y. Shoji F. (2020). Weakly-Supervised Learning for Lung Carcinoma Classification Using Deep Learning. Sci. Rep. 10 (1), 9297. 10.1038/s41598-020-66333-x 32518413 PMC7283481

[B18] Li G. Jiang D. Zhou Y. Jiang G. Kong J. Manogaran G. (2019). Human Lesion Detection Method Based on Image Information and Brain Signal. IEEE Access 7, 11533–11542. 10.1109/access.2019.2891749

[B19] Li Y. Ping W. (2018). Cancer Metastasis Detection with Neural Conditional Random Field. ArXiv e-prints [Preprint]. Available at: https://arxiv.org/abs/1806.07064 (Accessed June 19, 2018).

[B20] Lim H. Devesa S. S. Sosa J. A. Check D. Kitahara C. M. (2017). Trends in Thyroid Cancer Incidence and Mortality in the United States, 1974-2013. JAMA 317 (13), 1338–1348. 10.1001/jama.2017.2719 28362912 PMC8216772

[B21] Liu Y. Krishna G. Mohammad N. George E. D. Timo K. Aleksey B. (2017). Detecting Cancer Metastases on Gigapixel Pathology Images. ArXiv e-prints [Preprint]. Available at: https://arxiv.org/abs/1703.02442 (Accessed March 8, 2017).

[B22] Liu Y. Kohlberger T. Norouzi M. Dahl G. E. Smith J. L. Mohtashamian A. (2019). Artificial Intelligence-Based Breast Cancer Nodal Metastasis Detection: Insights into the Black Box for Pathologists. Arch. Pathol. Lab. Med. 143 (7), 859–868. 10.5858/arpa.2018-0147-OA 30295070

[B23] Lloyd R. V. Osamura R. Y. Klöppel G. Rosai J. (2017). WHO Classification of Tumours: Pathology and Genetics of Tumours of Endocrine Organs. 4th ed. Lyon: IARC Press.

[B25] Ozgur M. Sylvia L. A. Martin J. B. Sally E. C. Steven H. Jonathan B. M. (2021). Protocol for the Examination of Specimens from Patients with Carcinomas of the Thyroid Gland. Version: 4.3.0.0 Protocol Posting Date: June 2021 CAP Laboratory Accreditation Program Protocol Required Use Date: March 2022. Northfield: College of American Pathologists.

[B26] Ronneberger O. Fischer P. Brox T. (2015). U-Net: Convolutional Networks for Biomedical Image Segmentation. German, Cham: Springer.

[B27] Rosario P. W. Mourão G. F. (2019). Noninvasive Follicular Thyroid Neoplasm with Papillary-Like Nuclear Features (NIFTP): A Review for Clinicians. Endocr. Relat. Cancer 26 (5), R259–R266. 10.1530/ERC-19-0048 30913533

[B28] Seib C. D. Sosa J. A. (2019). Evolving Understanding of the Epidemiology of Thyroid Cancer. Endocrinol. Metab. Clin. North. Am. 48 (1), 23–35. 10.1016/j.ecl.2018.10.002 30717905

[B29] Shirsat H. Zhou F. Chang J. C. Rekhtman N. Saqi A. Argyropoulos K. (2021). Bronchiolar Adenoma/Pulmonary Ciliated Muconodular Papillary Tumor. Am. J. Clin. Pathol. 155 (6), 832–844. 10.1093/ajcp/aqaa194 33313677

[B30] Siegel R. L. Miller K. D. Fuchs H. E. Jemal A. (2021). Cancer Statistics, 2021. CA A Cancer J. Clin. 71 (1), 7–33. 10.3322/caac.21654 33433946

[B31] Skrede O.-J. De Raedt S. Kleppe A. Hveem T. S. Liestøl K. Maddison J. (2020). Deep Learning for Prediction of Colorectal Cancer Outcome: A Discovery and Validation Study. Lancet 395 (10221), 350–360. 10.1016/S0140-6736(19)32998-8 32007170

[B32] Song Z. Zou S. Zhou W. Huang Y. Shao L. Yuan J. (2020). Clinically Applicable Histopathological Diagnosis System for Gastric Cancer Detection Using Deep Learning. Nat. Commun. 11 (1), 4294. 10.1038/s41467-020-18147-8 32855423 PMC7453200

[B33] Szegedy C. Vanhoucke V. Ioffe S. Shlens J. Wojna Z. (2016). “Rethinking the Inception Architecture for Computer Vision,” in 2016 IEEE Conference on Computer Vision and Pattern Recognition (CVPR), Las Vegas, NV, USA, 27-30 June 2016 (IEEE), 2818–2826.

[B34] Tian Y. Yang L. Wang W. Zhang J. Tang Q. Ji M. (2019). Computer-Aided Detection of Squamous Carcinoma of the Cervix in Whole Slide Images. ArXiv e-prints [Preprint]. Available at: https://arxiv.org/abs/1905.10959 (Accessed May 27, 2019).

[B35] Tuttle R. M. Ahuja S. Avram A. M. Bernet V. J. Bourguet P. Daniels G. H. (2019). Controversies, Consensus, and Collaboration in the Use of 131I Therapy in Differentiated Thyroid Cancer: A Joint Statement from the American Thyroid Association, the European Association of Nuclear Medicine, the Society of Nuclear Medicine and Molecular Imaging, and the European Thyroid Association. Thyroid 29 (4), 461–470. 10.1089/thy.2018.0597 30900516

[B36] Wang D. Aditya K. Rishab G. Humayun I. Andrew H. (2016). Deep Learning for Identifying Metastatic Breast Cancer. ArXiv e-prints [Preprint]. Available at: https://arxiv.org/abs/1606.05718 (Accessed June 18, 2016).

[B37] Webster J. D. Dunstan R. W. (2014). Whole-Slide Imaging and Automated Image Analysis: Considerations and Opportunities in the Practice of Pathology. Vet. Pathol. 51 (1), 211–223. 10.1177/0300985813503570 24091812

[B38] Wen S. Kurc T. M. Gao Y. Zhao T. Saltz J. H. Zhu W. (2017). A Methodology for Texture Feature-Based Quality Assessment in Nucleus Segmentation of Histopathology Image. J. Pathol. Inf. 8, 38. 10.4103/jpi.jpi_43_17 PMC5609357 28966837

[B39] Zhang S. Sun K. Zheng R. Zeng H. Wang S. Chen R. (2021). Cancer Incidence and Mortality in China, 2015. J. Natl. Cancer Cent. 1 (1), 2–11. 10.1016/j.jncc.2020.12.001 PMC11256613 39036787

[B40] Zhang Z. Chen P. Mcgough M. Xing F. Wang C. Bui M. (2019). Pathologist-Level Interpretable Whole-Slide Cancer Diagnosis with Deep Learning. Nat. Mach. Intell. 1 (1), 236–245. 10.1038/s42256-019-0052-1

[B41] Zhao L. Pang P. Zang L. Luo Y. Wang F. Yang G. (2019). Features and Trends of Thyroid Cancer in Patients with Thyroidectomies in Beijing, China between 1994 and 2015: A Retrospective Study. BMJ Open 9 (1), e023334. 10.1136/bmjopen-2018-023334 PMC6347868 30782703

[B42] Zheng R. Zhang S. Zeng H. Wang S. Sun K. Chen R. (2022). Cancer Incidence and Mortality in China, 2016. J. Natl. Cancer Cent. 2 (1), 1–9. 10.1016/j.jncc.2022.02.002 PMC11256658 39035212

