# Supplementary material for: Deep Learning-Based Recognition of Different Thyroid Cancer Categories Using Whole Frozen-Slide Images
Source: Front Bioeng Biotechnol. 2022 Jul 6;10:857377. doi: 10.3389/fbioe.2022.857377 (PMC9298848; doi:10.3389/fbioe.2022.857377)
Supplement: Supplementary file 1 [file Table1.DOCX]

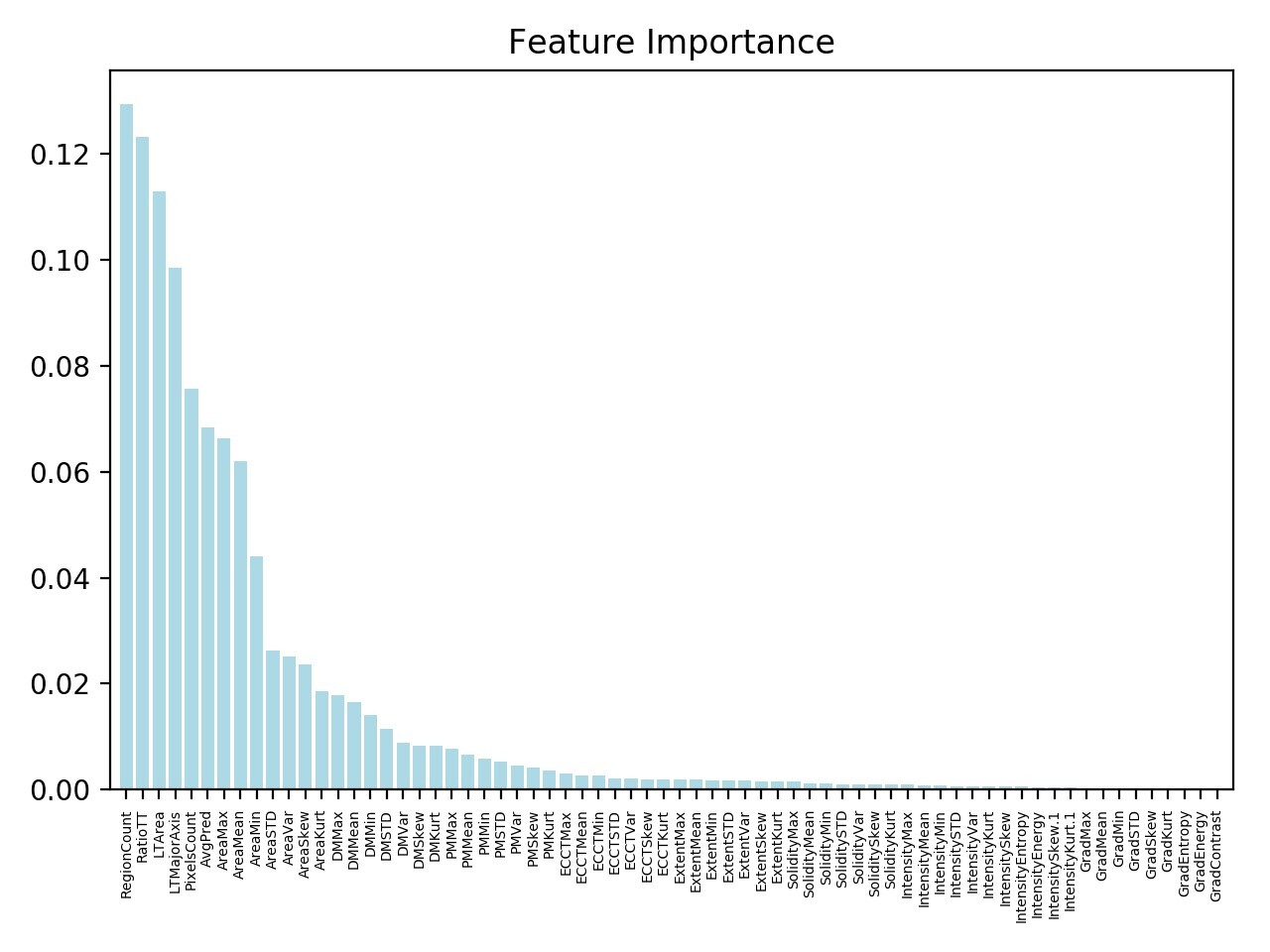


**Supplementary Figure 1**: Feature importance of the learned features for Random Forest classifier.

**Supplementary Table 1. Extracted Features**

| Category | Feature name | Description |
| --- | --- | --- |
| Geometric features | AreaMax, AreaMin, AreaMean, AreaSTD, AreaVar, AreaSkew, AreaKurt | The maximum, minimum, mean area of all connected regions; the standard deviation, variance, skewness, kurtosis of area of all connected region |
|  | PMMax, PMMin, PMMean, PMSTD, PMVar, PMSkew, PMKurt | The maximum, minimum, mean perimeter of all connected regions; the standard deviation, variance, skewness, kurtosis of perimeter of all connected region |
|  | DMMax, DMMin, DMMean, DMSTD, DMVar, DMSkew, DMKurt | The maximum, minimum, mean length of the major axis of all connected regions; the standard deviation, variance, skewness, kurtosis of the major axis of all connected region |
|  | ECCTMax, ECCTMin, ECCTMean, ECCTSTD, ECCTVar, ECCTSkew, ECCTKurt | The maximum, minimum, mean eccentricity of all connected regions; the standard deviation, variance, skewness, kurtosis of eccentricity of all connected region |
|  | ExtentMax, ExtentMin, ExtentMean, ExtentSTD, ExtentVar, ExtentSkew, ExtentKurt | The maximum, minimum, mean extent (ratio of pixels in the region to pixels in the total bounding box) of all connected regions; the standard deviation, variance, skewness, kurtosis of extent of all connected region |
|  | SolidityMax, SolidityMin, SoliditySTD, SolidityMean, SolidityVar, SoliditySkew, SolidityKurt | The maximum, minimum, mean solidity (ratio of pixels in the region to pixels in the convex hull image) of all connected regions; the standard deviation, variance, skewness, kurtosis of solidity of all connected region |
| Texture features | GradMax, GradMin, GradMean, GradSTD, GradVar, GradSkew, GradKurt, GradEntropy, GradEnergy, GradContrast, GradDissimilarity, GradHomogeneity, GradCorrelation | The maximum, minimum, mean, standard deviation, variance, skewness, kurtosis of the gradient channel value; the entropy, sum of squared elements, contrast, dissimilarity, homogeneity, correlation of the normalized co‑occurrence matrix of gradient channel value |
|  | IntensityMax, IntensityMin, IntensityMean, IntensitySTD, IntensityVar, IntensitySkew, IntensityKurt, IntensityEntropy, IntensityEnergy, IntensityContrast, IntensityDissimilary, IntensityHomogeneity, IntensityCorrelation | The maximum, minimum, mean, standard deviation, variance, skewness, kurtosis of the raw pixel value; the entropy, sum of squared elements, contrast, dissimilarity, homogeneity, correlation of the normalized co‑occurrence matrix of raw pixel value |
| Marginal features | CannyNonzero, CannyMean | Number of pixels with nonzero canny value; the mean of canny value |
| Other features | RegionCount | The number of the connected regions |
|  | RatioTT | The ratio of the tumor connected region to the tissue region |
|  | LTArea | The area of the largest connected region |
|  | LTMajorAxis | The major axis of the largest connected region |
|  | PixelsCount | The number of pixels with predicted probability larger than 0.9 |
|  | AvgPred | The average value of pixels in the tumor connected region |

**Supplementary Table 2. The top 30 important features can be summarized into 4 categories**

| Category | Features |
| --- | --- |
| Lesion count | RegionCount |
| Lesion area related | RatioTT, LTArea, PixelsCount, AvePred, AreaPred, AreaMean, AreaMax, AreaMin, AreaSTD, AreaVar, AreaSkew, AreaSkew |
| Lesion diameter related | DMMean, DMMax, DMMin, DMSTD, DMVar, DMSkew, DMSkew |
| Lesion perimeter(shape) related | PMMean, PMMax, PMMin, PMSTD, PMVar, PMSkew, PMSkew, ECCTMax, ECCTMean, TCCTMin |

**Supplementary Table 3. The Test 2 dataset was divided into training and test set for triple classification**

| Ground truth | Training | Test | Total |
| --- | --- | --- | --- |
| Malignant | 70 | 275 | 345 |
| Benign | 70 | 205 | 275 |
| Intermediate | 70 | 74 | 144 |
| Total | 210 | 554 | 764 |

**Supplementary Table 4. Four grid table of our framework and pathologist**

|  | Correctly diagnosed | Misdiagnosed | Total |
| --- | --- | --- | --- |
| Our framework | 492 | 17 | 509 |
| Pathologist | 501 | 8 | 509 |

**Supplementary Table 5. Result evaluation metrics for our UNet and random forest model**

| dataset | Sensitivity | Specificity | PPV | NPV | AUC |
| --- | --- | --- | --- | --- | --- |
| Test 1 | 0.954 | 0.973 | 0.968 | 0.962 | 0.986 |
| Test 2 | 0.955 | 0.845 | 0.778 | 0.969 | 0.946 |
